# Supplementary material for: Motor-driven advection competes with crowding to drive spatiotemporally heterogeneous transport in cytoskeleton composites
Source: Front Phys. Author manuscript; Available in PMC 2023 Aug 4. (PMC10403238; doi:10.3389/fphy.2022.1055441)
Supplement: SI [file NIHMS1905677-supplement-SI.pdf]

Motor-driven advection competes with crowding to drive spatiotemporally heterogeneous transport in cytoskeleton composites

Janet Y. Sheung[1,2*], Jonathan Garamella[3], Stella K. Kahl[1], Brian Y. Lee[2], Aaron Xie[2], Ryan J McGorty[3], Rae M Robertson-Anderson[3]

[1]W. M. Keck Science Department, Scripps College, Claremont, CA, USA
[2]W. M. Keck Science Department, Claremont McKenna College, Claremont, CA, USA
[3]Physics and Biophysics Department, University of San Diego, San Diego, CA, USA

Supplementary Material

1 Supplementary Figures and Tables

Figure S1: $\tau(q)$ versus wavevector q evaluated over the entire q range over which corresponding $D(q,\Delta t)$ curves are fit.

Figure S2: Stretching exponents $\gamma(q)$ determined from fits to $D(q,\Delta t)$ and plotted for all q values used to determine the power-law dependence of $\tau(q)$.

Figure S3: van Hove distributions of particle displacements in the x- and y- directions for all composites.

Figure S4: van Hove distributions for 11 individual measurements for $\phi_A = 1$.

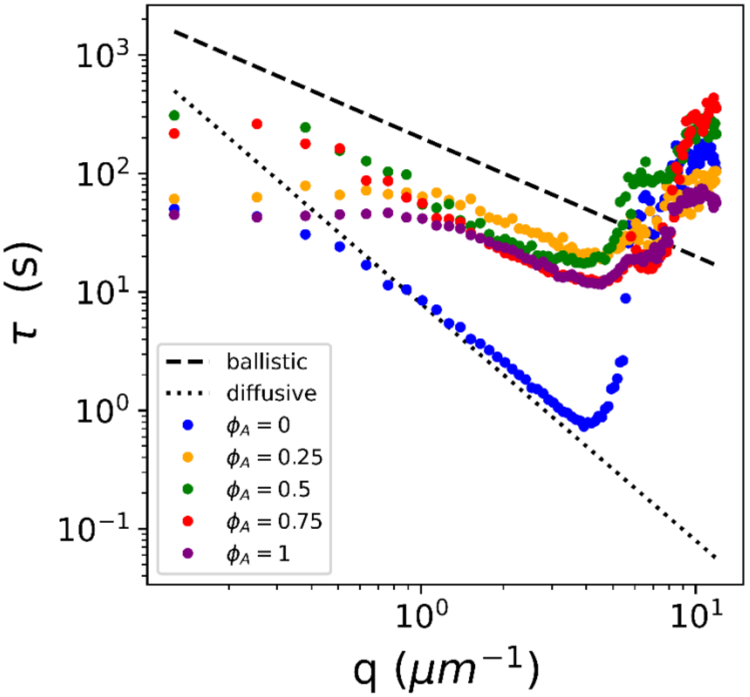

Figure S1: $\tau(q)$ versus wavevector q evaluated over the entire q range over which corresponding $D(q, \Delta t)$ curves are fit. Data shown is the same as in Fig 5B but for an extended range $q = 0.16 - 16\ \mu\text{m}^{-1}$. Dashed and dotted lines correspond to ballistic and diffusive scaling exponents $\beta = 1$ and 2, respectively. To determine scaling behavior of composites, we evaluate $\tau(q)$ for $q = 1 - 3.9\ \mu\text{m}^{-1}$ over which power-law behavior is observed for all composites. The non-physical upticks in $\tau(q)$ for $q > 3.9\ \mu\text{m}^{-1}$ are due to the optical resolution of our microscope. While the theoretical resolution limit is $q \simeq 10\ \mu\text{m}^{-1}$ with an objective of NA=1.0, non-ideal imaging conditions, such as imaging across a capillary tube which has a refractive index not perfectly matched to that of the sample, reduces this limit to ~4 μm^{-1} in our setup. In the low-q limit, the unphysical rollovers and plateaus in some of the data are due to a combination of the image size, the maximum lag time we probe, and noise. We analyze 256×256 square-pixel images with a pixel size of ~0.1 μm, setting a minimum of $q \simeq 2\ \mu\text{m}^{-1}$. However, we are further limited in certain cases by the accessible time scales. Namely, density fluctuations at small q values are expected to slowly decay, and the maximum Δt over which we fit $D(q, \Delta t)$ is ~100 s, above which the data is prohibitively noisy to accurately fit due to low statistics.

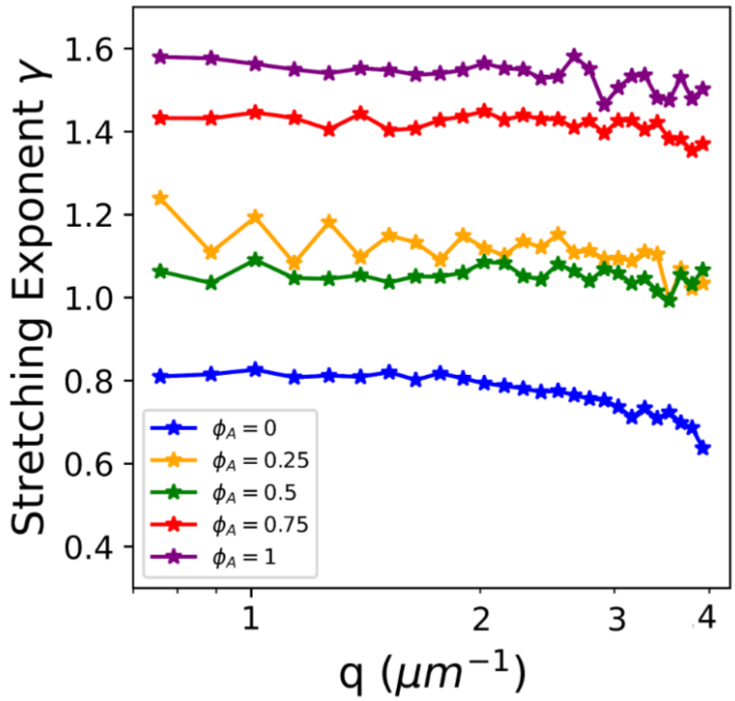

Figure S2: Stretching exponents $\boldsymbol{\gamma(q)}$ determined from fits to $\boldsymbol{D(q, \Delta t)}$ and plotted for all $\boldsymbol{q}$ values used to determine the power-law dependence of $\boldsymbol{\tau(q)}$. $\gamma(q)$ values for all composites are approximately q-independent, validating our power-law analysis of $\tau(q)$. Averaging over q for each composite yields the data shown in Fig 5G.

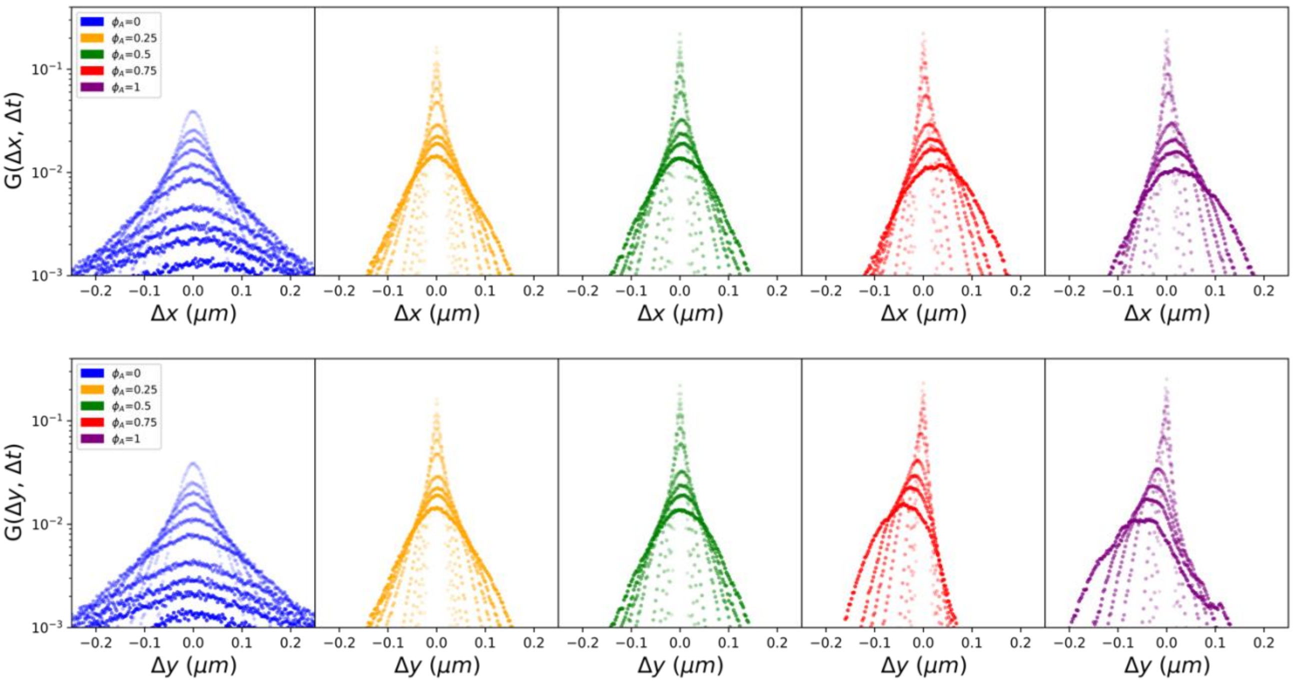

Figure S3: van Hove distributions of particle displacements in the x- and y- directions for all composites. van Hove distributions $G(\Delta x, \Delta t)$ (top) and $G(\Delta y, \Delta t)$ (bottom) of particle displacements Δx and Δy, measured via SPT, for lag times $\Delta t = 0.1, 0.2, 0.3, 0.5, 1, 2, 3, 5, 10, 15$ s denoted by the color gradient going from light to dark for increasing Δt. Each panel corresponds to a different composite demarked by their ϕ_A value with color-coding as in Fig 3. Data shown is the same as that in Fig 3A separated into x- and y- direction distributions. For reference, x- and y- directions correspond to the narrow and long dimensions of the capillary sample chamber, respectively.

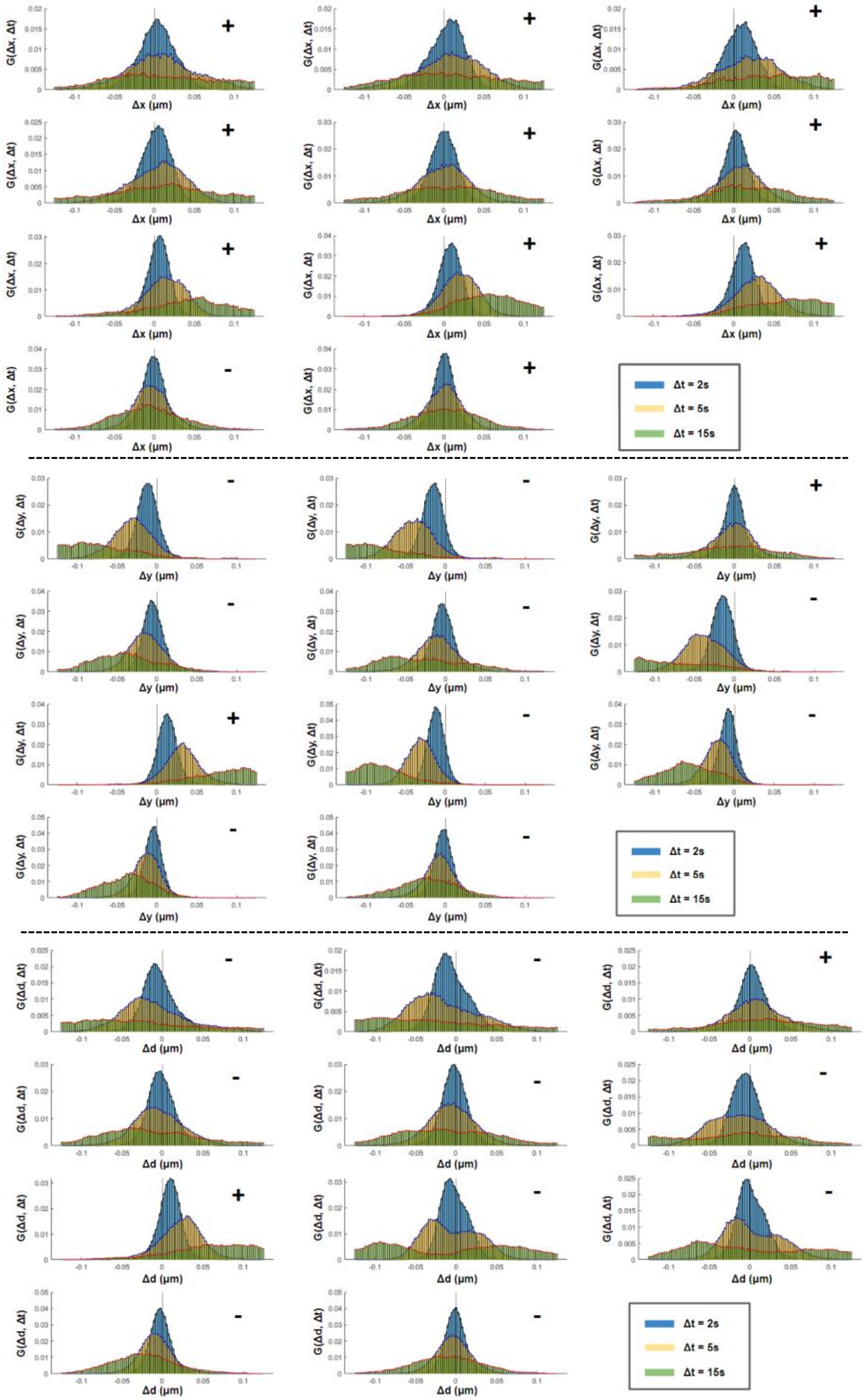

Figure S4: van Hove distributions for 11 individual measurements for $\boldsymbol{\phi_A = 1}$. van Hove probability distributions $G(\Delta x, \Delta t)$, $G(\Delta y, \Delta t)$, and $G(\Delta d, \Delta t)$ (from top to bottom) for particle displacements $\Delta x, \Delta y$, $\Delta d = \Delta x \cup \Delta y$ for each measurement of the $\phi_A = 1$ composite. Each plot displays distributions for $\Delta t = 2, 5, 15\ s$ with the dashed vertical line demarking zero displacement. The net direction of motion for each trial, positive or negative, is indicated in the upper right as + or -. $G(\Delta x, \Delta t)$ and $G(\Delta y, \Delta t)$ distributions are primarily in the positive and negative directions, respectively, with $G(\Delta y, \Delta t)$ distributions displaying relatively larger deviations from zero.
